# Exposure time as an influencing factor among rheumatoid arthritis patients subjected to traditional Siwan therapy

**DOI:** 10.1097/MD.0000000000035105

**Published:** 2023-09-15

**Authors:** Noha F. Mahmoud, Howida A. Fouda, Islam I. Omara, Nashwa M. Allam

**Affiliations:** a Department of Rehabilitation Sciences, College of Health and Rehabilitation Sciences, Princess Nourah bint Abdulrahman University, Riyadh, Saudi Arabia; b Department of Physical Therapy for Internal Diseases, Faculty of Physical Therapy, 6 October University, Giza, Egypt; c Department of Animal Production (Nutrition Division), Faculty of Agriculture, Cairo University, Giza, Egypt; d Department of Orthopedics and Orthopedic Surgery, Faculty of Physical Therapy, Ahram Canadian University, Giza, Egypt.

**Keywords:** central sensitization, pain perception, psammotherapy, rheumatoid arthritis, Siwan traditional therapy

## Abstract

Rheumatoid arthritis (RA) is a long-term autoimmune disease characterized by intra- and extra-articular manifestations. Sand therapy is traditionally indicated for RA, chronic pain, skin diseases, and musculoskeletal disorders. Many places in the world use sand therapy, including Siwa, which is a famous place in Egypt. This study investigated the exposure time to Siwan traditional therapy as a factor influencing central sensitization, pain severity, pain threshold, and kinesiophobia in RA by measuring the central sensory inventory (CSI), visual analogue scale, pressure algometer, and TAMPA kinesiophobia scale, respectively. Twenty-four patients with RA were recruited from 6 traditional healing centers, 24 RA patients were recruited and randomly assigned to 2 equal groups (GI and GII). The first received Siwan traditional therapy for 3 days, while the second received the same program for 5 days. The results revealed a significant difference in CSI between pre- and posttreatment within the GII (*P* = .038). The Tampa Scale score improved significantly in both groups (*P* = .004 and *P* = .014, respectively). Pain severity and pain threshold at all sites showed significant posttreatment improvements in the GII. Significant posttreatment changes were only found for GI in terms of pain severity and the most painful joint (*P* = .010 and *P* = .035, respectively). Significant changes were observed in kinesiophobia, pain severity, and pain threshold in the most painful joint 3 and 5 days after Siwan traditional therapy. Despite the nonsignificant differences in all parameters between the 2 groups, all the measured parameters produced favorable results after 5 days of treatment, suggesting the need for a long-term effect investigation.

## 1. Introduction

The Siwa is an oasis located in the western desert of Egypt. It covers an area of 7500 km^2^ area. The climate is extremely dry for almost 6 months of the year. The maximum precipitation is 9 mm in 1 month of the year. Precipitation is 2 mm for the rest of the year.^[1]^ The isolation and unique climate of Siwa were preserving factors for some traditional old therapies like psammotherapy, which is an old practice extending back to the period of Roman civilization.^[2]^ Traditionally, it has been used in the form of hot sand baths, mostly heated by the sun, for therapeutic purposes.^[3]^ This depends on the traditions of the people living in that place and the availability of natural resources such as sand.^[2]^ Even today, several countries are still using sand baths. Examples include Portugal,^[4]^ Japan,^[5]^ Egypt,^[6]^ and many other countries.^[7–9]^ Psammotherapy is commonly indicated for osteoarthritis, posttraumatic injuries, osteoporosis, gout, fibromyalgia, and chronic rheumatic conditions.^[10]^

Rheumatoid arthritis (RA) is a long-term autoimmune disease characterized by intra- and extra-articular manifestations. RA is thought to affect between 0.5% and 1% of the population worldwide.^[11]^ RA also affects other systems in the body through its immune-mediated inflammatory effect, characterized by spontaneous and symmetrical pain in the affected joints due to inflammation. Joint stiffness, particularly after immobility, such as after sleeping, is another characteristic of the disease. Movement within normal ranges and pressure at the joint margins increase pain severity.^[12,13]^ However, mild exercise, low humidity, and warming of the affected area improved the symptoms of RA.^[14]^

Pain is the primary trigger for seeking medical care. Patients with chronic pain visit their primary care clinician more often. Pain evaluation also helps determine the modifications needed in the patient’s treatment plan. However, pain is a subjective variable and is very difficult to measure.^[15]^ Traditionally, rheumatic pain is assumed to be directly related to peripheral inflammation; therefore, physicians have considered it an inflammatory marker. Disease-modifying antirheumatic drugs have been reported to significantly decrease pain. However, persistent pain in many patients has been reported, even with treatment.^[16]^ Additionally, a difference between the inflammation assessed by physicians and patient-reported pain has been reported in other studies.^[17,18]^ Chronic pain differs from acute pain in that it typically lasts longer and is more likely to be influenced by input from the central nervous system (CNS), whereas acute pain is frequently caused predominantly by inflammation and/or damage to peripheral tissues (i.e., nociceptive input).^[19]^

Consistently elevated nociceptive impulse activity, similar to that observed in RA, may cause peripheral and central sensitization, elevated sympathetic nervous system activity, and disruption of endogenous inhibitory systems.^[20,21]^ The mechanisms for experiencing pain are not fixed but rather dynamic. The term “plasticity of the nervous system” is used to describe the innate ability of the nervous system to change and adapt. Damage to peripheral tissues can trigger a plastic response in the CNS, which can extend pathological pain.^[22]^

Changes in both cells and their associated neurotransmitters are what we mean when discussing plasticity.^[23]^ The phenotype of neurons can be altered such that they transmit pain signals instead of pressure or touch signals.^[24]^ New sprouting of the dorsal horn may result in an expanded receptive field or formation of new synapses.^[25]^ The number of pain-transmitting receptors on the cell surface may either increase or decrease. The threshold at which neurons fire may change, and there may be disruptions in the circuit that control pain amplification or pain inhibition.^[26,27]^ This activity occurs continuously in both the peripheral nervous system and CNS.

In many musculoskeletal disorders, pain-related fear of movement or kinesiophobia plays a major role in the onset, persistence, and exacerbation of chronic disability.^[28]^ A better understanding of how pain, kinesiophobia, and physical function performance are related will be important for developing new ways to treat diseases and educate patients and their families.^[29]^

RA usually requires long-term treatment, with associated risks of drug toxicity.^[30]^ Efficacy, safety, route of administration, and treatment costs are important determinants in the choice of treatment.^[31]^ Although different treatment plans and monitoring can help patients achieve early and long-lasting clinical and radiographic remission, the high cost of drugs and limited healthcare budgets make it difficult for people to receive RA treatment. The high cost of treatment, both directly and indirectly, makes it difficult for patients to pay their bills and lower their quality of life.^[32]^ Increasingly, patients are visiting rheumatologists who expect them to treat pain as a discrete symptom. Currently, pain treatment is more holistic, considering the patient’s biopsychosocial needs rather than only the patient’s physical needs.^[33]^ Egypt is a developing country with limited financial resources for both individuals and the healthcare system. RA is a chronic disease that presents a heavy burden on the shoulders of patients. Therefore, many patients with RA prefer traditional medicines. They consider it a healing experience in a way that many patients plan for annually. From this point of view, traditional Siwan therapy is cheaper, safer, and more effective. Traditional healers in Siwa suggest the duration of treatment according to their experience, which depends mostly on their history of estimated improvement. However, it is the patient’s decision to accept or refuse this suggestion according to his tolerance and/or financial status. Healers traditionally suggest 3, 5, or 7 hot sand baths for RA once a day. To the best of our knowledge, only 3 previous studies have been performed in Siwa and proved the effect of Siwan traditional therapy on RA.^[6,34,35]^ The fourth study investigated some of the risk factors associated with hot sand.^[36]^ None of the previous studies have discussed the different factors affecting the treatment protocols followed in the Siwa. Pain is the most common complaint in patients with RA. The current study is a baseline for a future cohort study that aims to establish the effect of such therapy. This helps determine whether the number of treatment sessions is a variable affecting treatment outcomes. It also provides information about pain intensity, kinesiophobia, and pain threshold changes due to hot sand.

## 2. Materials and methods

### 2.1. Study design and sample

**Figure 1.**
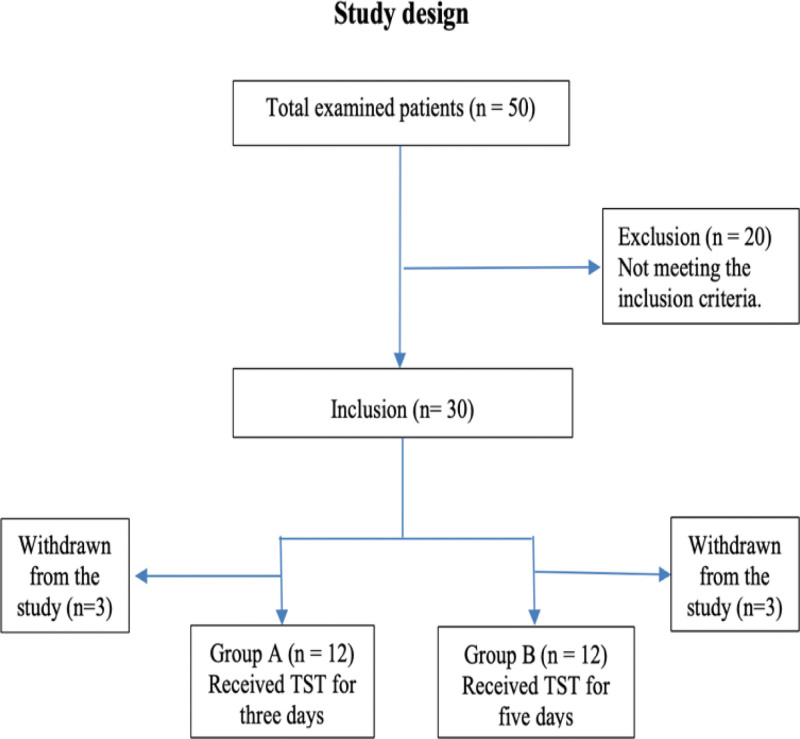
Study design, only 30 patients out of 50 met the inclusion criteria. Only 24 patients were included in the final analysis.

Traditional Siwan therapy is performed only for 3 month a year, starting from mid-June until the end of the first week of September. In the current observational cross-sectional study, 50 patients with RA were recruited from 6 traditional healing centers in Siwa. Thirty patients met the inclusion criteria. However, 24 patients (n = 24) were included in the final analysis. Eligible individuals were diagnosed with RA according to the 2010 American College of Rheumatology/European Leaguen Against Rheumatism classification criteria.^[30]^ They were assigned to 2 groups. Group (I) consisted of 15 patients who received Siwan therapy for 3 days; and group (II) consisted of 15 patients who received the same treatment for 5 days. The patients’ ages ranged from 30:60 years, and all had chronic pain (> 6 months). Before the study, patients were on stable doses of disease-modifying antirheumatic drugs for 6 weeks and nonsteroidal anti-inflammatory drugs for 2 weeks. Patients were required to have a pain score between 30 and 70 on the present pain intensity scale. Corticosteroid injections were not allowed during the 4 weeks prior to the study. Informed consent was obtained from all the subjects involved in the study. Three patients did not finish the treatment. They traveled back home for personal reasons. Two patients traveled home after treatment before the final assessment. One patient finished her treatment but refused to continue in the research second assessment with no specific reason. Finally, Group (I) consisted of 12 patients who received Siwan therapy for 3 days; and group (II) consisted of 12 patients who received the same treatment for 5 days as shown in Figure 1. Patients who met the following exclusion criteria were excluded: uncontrolled arterial hypertension, a history of renal transplantation, coronary heart disease, pregnancy, or any bleeding disorder. This study was carried out in accordance with the Helsinki Declaration guidelines and was approved by the Ethics Committee of Cairo University’s Faculty of Physical Therapy, Egypt (P.T.REC/012/00947).

### 2.2. Sample size effect

This study’s sample size was determined using G*power 3.1.9 (G power program version 3.1, Heinrich-Heine-University, Düsseldorf, Germany) for two-tailed test. Sample size calculation was based on *t*-tests (means: difference between 2 independent means-two groups), type I error (alpha = 0.05), type II error (1-βeta = 90%), and effect size d = 1.42857 for the main variable outcomes. Considering a 15% drop out rate, the appropriate sample size effect or this study included 24 patients (12 patients per group).

### 2.3. Outcomes

• Pain severity was measured using a Visual Analogue Scale: a Visual Analogue Scale is typically a horizontal line 100 mm long that the patient marks to represent his perception of the current pain status.^[37]^
• The Arabic version of the Central Sensitization Inventory was used to measure central sensitization. CSI is a tool that helps people identify signs of nociplastic pain.^[38]^ It was developed as a comprehensive screening tool with high reliability to determine whether a CS component exists or not.^[39,40]^ There are 2 parts to CSI. Part A, which is used to measure CS symptoms, is made up of 25 self-reported questions about physical and emotional symptoms. Each question was scored from 0 to 100, with 0 being the best score and 100 being the worst. A 5-point Likert scale (4 = always, 3 = often, 2 = sometimes, 1 = rarely, 0 = never) was used to rate each item. Part B checked for a history of specific disorders, including 7 different CS syndromes (e.g., fibromyalgia, irritable bowel syndrome, and restless leg syndrome). There is strong evidence that CS is involved in these 7 disorders. The CSI Part A score was divided into 5 groups based on the severity of the condition: subclinical (scores between 0 to 29), mild (30 to 39), moderate (40 to 49), severe (50 to 59), and extreme (scores between 60 to 100).^[41]^
• Kinesiophobia was measured using the Arabic version of the Tampa kinesiophobia scale to assess fear of reinjury associated with physical movement. Respondents rated 17 items on a 4-point scale ranging from (1 “strongly disagree” to 4 “strongly agree”). The total score ranges from 17 to 68, with higher scores indicating greater fear of movement or reinjury.^[42]^
• Pain thresholds were measured using an algometer at the third metacarpophalangeal metacarpophalangeals (MCPs) joints, wrist, and most inflamed joint.^[43]^ Pressure is one of the most common stimuli used to test pain thresholds in RA because it is thought to be the most reflective of arthritic pain.^[44]^

### 2.4. Intervention

The most beneficial time for a sand bath is during the summer months in June, July, and August, as the sun is very hot. Sand bathing was performed in the afternoon at 1:4 pm When the air temperature was 40:45 °C. Traditional healers usually dug a shallow hole in the sand, with dimensions of 20:40 cm deep, 80 cm wide, and 1:2 m in length. The hole is dug in the early morning at 10 am until the time of the session for heating the sand using sun rays. Before starting the sand bath, patients were instructed to avoid applying topical cream or lotion. They were also notified that this intervention would leave their body dusty and that they were not allowed to bath until the end of the 3 or 5 sessions.

During the sand bath, the patient was asked to lay down in the hole and was covered to the neck level with hot dry sand from the surface of the desert which is 75 to 82 °C in temperature. This takes approximately 10 to 15 minutes or according to the patient’s tolerance, but not more than 30 minutes. When the sand became wet from perspiration, it was replaced with hot-dry sand. Ten to 20 cm under the sand surface, the sand temperature was 50 to 60 °C. Immediately after the sand bath, the patient was wrapped carefully in a blanket and placed in a well-sealed tent located close to the hole for 15 minutes or according to patient tolerance. In the tent, the patient’s body relaxed and returned to its normal condition. Wrapping protects the patient from cold air drafts, which can lead to adverse effects, such as muscle soreness and headache. Patients were provided with many hot liquids (e.g., lemon juice, paddock, anise, and licorice) to prevent dehydration. Drinking water is prohibited immediately after the sand bath. Patients were instructed to continue drinking fluids until their bodies were replenished with dehydration. The patients were instructed to lie down until the body was adjusted to the lower environmental temperature. They were also asked to wipe the sand off their body and change their clothes when sweating on their shirt dries. Hot water was used to wash hands and face. water anywhere else in their bodies is avoided. Cold, fans, and air conditioners are prohibited during treatment days. On the final day of the sand baths, the traditional healer gave the patient a full-body massage using olive oil.

## 3. Results

### 3.1. Statistical analysis

Data were examined using the normality assumption test and homogeneity of variance. Normality test of the data using the Shapiro–Wilk test was used, which indicated that the data were not normally distributed (*P* < .05) after removing outliers that were detected by box and whisker plots. Additionally, Levene test for the homogeneity of variance revealed a significant difference (*P* < .05). These findings allowed us to conduct both parametric and nonparametric analyses. The data were not normally distributed, and nonparametric analyses were performed. Statistical analysis was conducted using the 25th version of the Statistical Package for the Social Sciences (SPSS Inc., Chicago, IL). Quantitative data are expressed as means and standard deviations, and qualitative data are expressed as numbers and percentages. The Wilcoxon signed-rank test was used to compare pre- and posttreatment within 3-days group and 5-days group for pain severity and pain threshold. The Mann–Whitney *U* test was used to compare GI and GII pre- and posttreatment for pain severity and pressure algometer variables. The chi-square test was used to compare CSI and Tampa variables between the groups. All statistical analyses were significant at the level of significance (*P* ≤ .05).

### 3.2. Results

In the current study, 24 rheumatoid patients from both sexes (5 males and 19 females) were randomly assigned to 2 groups (12 patients/group). There were no significant differences (*P* > .05) in patient demographic data for age (*P* = .116), disease duration (*P* = .248), gender (*P* = .615), academic level (*P* = .799), medication history (*P* = .414), or Siwan traditional therapy history (*P* = 1.000) between the 3-days group (G I) and 5-days groups (G II) (Table 1).

**Table 1:** Patient’s general characteristics in 3-day group and 5-day group.

Items		Groups		*P*-value
		3-days group (n = 12) G1	5-days group (n = 12) GII	
Age (year)		41.17 ± 14.39	51.64 ± 14.89	.116
Date of duration		6.56 ± 1.08	13.50 ± 2.56	.248
Gender	Males	3 (25.00%)	2 (16.70%)	.615
	Females	9 (75.00%)	10 (83.30%)	
Academic level	None educated	2 (16.70%)	4 (33.30%)	.799
	Primary educated	3 (25.00%)	3 (25.00%)	
	Secondary educated	3 (25.00%)	2 (16.70%)	
	Higher educated	4 (33.30%)	3 (25.00%)	
Medication	Medicated	5 (41.70%)	7 (58.30%)	.414
	Non-medicated	7 (58.30%)	5 (41.70%)	
Siwan traditional therap previous practice	Yes	4 (33.30%)	4 (33.30%)	1.000
	No	8 (66.70%)	8 (66.70%)	

Quantitative data (age and disease duration) were expressed as mean ± standard deviation and compared using the Mann–Whitney *U* test.

Qualitative data (gender, academic level, medication, and Siwan therapy previous practice) are expressed as numbers (percentages) and compared using the chi-square test.

NS = nonsignificant, *P*-value = probability value.

The statistical analysis (time effect) for CSI and Tampa Scale variables within each group (Table 2) revealed no significant difference in CSI (*P* = .588; *P* > .05) between pre- and posttreatment within 3-days group. However, there was a significant difference in CSI (*P* = .038; *P* < .05) between pre- and posttreatment within 5-days group. The Tampa Scale distribution was significantly affected (*P* > .05) by time within the 3-days group (0.014) and 5-days group (*P* = .004).

**Table 2:** Within and between groups comparison for CSI and Tampa Scale variables.

Variables		Categories	Groups		*P*-value
			3-days group (n = 12) G1	5-days group (n = 12) GII	
CSI	Pretreatment	Subclinical	1 (8.30%)	2 (16.70%)	.675
		Mild	2 (16.70%)	1 (8.30%)	
		Moderate	4 (33.30%)	2 (16.70%)	
		Severe	1 (8.30%)	3 (25.00%)	
		Extreme	4 (33.30%)	4 (33.30%)	
	Posttreatment	Subclinical	3 (25.00%)	9 (75.00%)	.099
		Mild	4 (33.30%)	1 (8.30%)	
		Moderate	2 (16.70%)	0 (0.00%)	
		Severe	1 (8.30%)	0 (0.00%)	
		Extreme	2 (16.70%)	2 (16.70%)	
	*P*-value		0.588	0.038*	
Tampa Scale	Pretreatment	Low	3 (25.00%)	2 (16.70%)	.615
		High	9 (75.00%)	10 (83.30%)	
	Posttreatment	Low	9 (75.00%)	9 (75.00%)	1.000
		High	3 (25.00%)	3 (25.00%)	
	*P*-value		0.014*	0.004*	

Data are expressed as mean ± standard deviation (SD).

*P*-value = probability value.

* Significant (*P* < .05).

There was no significant difference between the 3-days group and 5-days group in CSI at pretreatment (*P* = .675; *P* > .05) and posttreatment (*P* = .099; *P* > .05) due to the group effect (Table 2). Moreover, Tampa levels did not differ significantly between GI and GII at pretreatment (*P* = .615) and posttreatment (*P* = 1.000).

Pairwise comparison tests (time effect) for pain severity and pressure algometer variables within each group (Table 3) showed that there was a significant (*P* < .05) decrease in pain severity and increased pain threshold in the most painful joint posttreatment compared with pretreatment within GI (*P* = .010 and *P* = .035, respectively) and GII (*P* = .018 and *P* = .021, respectively). There were no significant differences between pre- and posttreatment within the GI in the right pressure pain threshold for the 3rd MCP (*P* = .182) and wrist (*P* = .530). However, there was a significant (*P* < .05) increase in the 3rd MCP (*P* = .034) and wrist (*P* = .003) posttreatment compared with pretreatment in GII. In the GI group, the left pain threshold did not differ significantly (*P* > .05) between pre-and posttreatment for 3rd MCP (*P* = .084) and wrist (*P* = .182). In contrast, the time effect in the 5-days group had a significantly (*P* < .05) higher left pain threshold of 3rd MCP (*P* = .005) and wrist (*P* = .012) posttreatment than pretreatment. These significant decreases in pain severity and increases in pain threshold in the 3rd MCP, wrist, and most pain posttreatment were more favorable of rheumatoid patients in the 5-days group than 3-days group. Moreover, the 5-days group improved higher decrease in pain severity and increased pain threshold in the most painful, right 3rd MCP, right wrist, left 3rd MCP, and left wrist (29.77%, 87.62%, 38.82%, 58.02%, 50.93%, and 46.28%, respectively) than those in the 3-days group (29.28%, 37.99%, 31.09%, 28.37%, 41.94%, and 30.72%, respectively). Pairwise comparison tests (group effect) for pain severity and pain threshold (right and left) variables between both groups (Table 3) indicated no significant differences (*P* > .05) in the pretreatment of pain severity (*P* = .396), pain threshold in the most painful (*P* = .332), right 3rd MCP (*P* = .065), right wrist (*P* = .054), left 3rd MCP (*P* = .910), and left wrist (*P* = .159) between the 3-days group and 5-days group. The same trend (*P* > .05) was observed posttreatment for pain severity (*P* = .581) and pain threshold in the most painful (*P* = .684), right 3rd MCP (*P* = .261), right wrist (*P* = .952), left 3rd MCP (*P* = .785), and left wrist (*P* = .412) between the 3-days group and 5-days group.

**Table 3:** Within and between groups comparison for pain severity and pressure algometer variables.

Variables	Items	Groups (mean ± SD)		Change	*P*-value
		3-days group (n = 12) GI	5-days group (n = 12) GII		
Pain severity	Pretreatment	64.58 ± 31.83	73.33 ± 29.02	8.75	.396
	Posttreatment	45.67 ± 25.27	51.50 ± 31.48	5.83	.581
	Change	18.91	21.83		
	Improvement %	29.28%	29.77%		
	*P*-value	0.010*	0.018*		
Most painful joint	Pretreatment	1.79 ± 1.38	1.05 ± 0.85	0.74	.332
	Posttreatment	2.47 ± 1.20	1.97 ± 1.13	0.50	.684
	Change	0.68	0.92		
	Improvement %	37.99%	87.62%		
	*P*-value	0.035*	0.021*		
Right 3rd MCP	Pretreatment	1.93 ± 0.41	1.52 ± 0.66	0.41	.065
	Posttreatment	2.53 ± 0.26	2.11 ± 0.40	0.42	.261
	Change	0.60	0.59		
	Improvement %	31.09%	38.82%		
	*P*-value	0.182	0.034*		
Right wrist	Pretreatment	2.15 ± 0.51	1.62 ± 0.70	0.53	.054
	Posttreatment	2.76 ± 1.77	2.56 ± 0.38	0.20	.952
	Change	0.61	0.94		
	Improvement %	28.37%	58.02%		
	*P*-value	0.530	0.003^*^		
Left 3rd MCP	Pretreatment	2.17 ± 0.33	1.61 ± 0.56	0.56	.910
	Posttreatment	3.08 ± 1.54	2.43 ± 0.73	0.65	.785
	Change	0.91	0.82		
	Improvement %	41.94%	50.93%		
	*P*-value	0.084	0.005*		
Left wrist	Pretreatment	2.93 ± 0.25	1.88 ± 0.90	1.05	.159
	Posttreatment	3.83 ± 2.51	2.75 ± 0.75	1.08	.412
	Change	0.90	0.87		
	Improvement %	30.72%	46.28%		
	*P*-value	0.182	0.021*		

Data are expressed as mean ± standard deviation (SD).

*P*-value = probability value.

* Significant (*P* < .05).

## 4. Discussion

One in 4 people around the world has chronic pain conditions, and many of them have musculoskeletal causes.^[45]^ Although sand baths have been used to treat RA for centuries, there has not been much scientific investigation of their efficacy. There is also a lack of a perfect choice of treatment dose for each pathological condition in traditional Siwan therapy. The purpose of this observational cross-sectional study was to determine the efficacy of such therapy in reducing pain, pain threshold, central pain, and kinesiophobia in patients with RA. It also investigated whether exposure time can affect treatment outcomes by comparing the 3-day group to the 5-day group. The current study showed that central pain in both groups improved significantly in GII (5-day group) but not in GI (3-day group). Additionally, in both groups, a significant decrease in pain severity was found as well as a significant increase in pain threshold at the most painful joint. When comparing the 2 groups, there were no significant difference. Regarding the pain threshold at the 3rd MCP and wrist, nonsignificant improvement was found in GI, while significant improvement was found in GII.

Chronic inflammatory pain arises when inflammatory mediators are not neutralized, and the overexpression of proinflammatory cytokines and chemokines leads to peripheral and central sensitization.^[46]^ Central sensitization syndrome was present in 41% of the patients with RA.^[47]^ A longer duration of symptoms is associated with a lower pressure pain threshold, suggesting that centrally mediated mechanisms evolve over time.

Changes in pain intensity, central pain, and pain threshold can be attributed to the hyperthermia effect of heated sand on the skin’s receptors and vessels. There is a complex communication network between the spinal cord, CNS, immune-endocrine system, and the skin is a key neuroendocrine organ that synthesizes and secretes numerous neurotransmitters and peptide substances that modulate inflammation, immune responses during host defense, pain, and pruritus.^[48]^

Transient receptor potential (TRP), a protein receptor dispersed widely in the body and skin, can be activated by various forms of thermal stimuli.^[49]^ TRPV1, a member of the TRP vanilloid family, is activated by inflammatory mediators that induce hyperalgesia^[50]^ and thermal stimulation at temperatures >43°C^[51]^ which are achieved by the hot sands of Siwa. Many studies refer to TRPV1 role in modulating pain conversion, regulating brain synaptic transmission and plasticity. TRPV1 is effective in several neurological and psychiatric disorders, including depression, anxiety, and depression.^[52–54]^

When TRPV1 receptors are continuously activated, a large and sustained increase in intracellular Ca2 + levels is induced, resulting in excitotoxicity that compromises and deletes TRPV1-expressing cells.^[55]^ As a result, in hyperalgesic conditions, such as inflammatory or neuropathic pain, overstimulation of the TRPV1 receptor would be useful in deleting TRPV1 receptor-positive neurons and eradicating sensitivity to nociceptive stimuli without disrupting normal sensory transmission via fibers that do not express TRPV1 receptors.^[56]^

Decreased pain severity and increased threshold can also be attributed to the thermal effect of a sand bath on circulation, as an abnormally low level of peripheral circulation is one of the causes of pain.^[57]^ In a previous study on the effect of Uyghur sand therapy on the dynamics of arterial flow in knee joints,^[7]^ a sand bath increased the inner diameter of knee arteries, sped up blood flow, and helped to increase the uniform velocity distribution. Besides. In that study, the sand bath was only applied to the knee, whereas in the current study, the Siwan sand bath was applied to the entire body, resulting in improved circulation in all body joints. Therefore, exposure to more thermal stimuli is better than less thermal stimuli, which may explain the significant changes of pain in the group received 5 days of treatment compared to those received 3 days of treatment.

As expected, the reduction in pain intensity and increase in pain threshold and their beneficial effect on anxiety significantly alleviated kinesiophobia in both groups. This may be due to the pain reduction achieved, but it could also be due to the psychological support provided by the patients’ conversations. Owing to limited resources, the multidisciplinary treatment approach in Siwa is not the standard medical practice for outpatient rheumatic patients in Egypt. However, in traditional Siwan healing centers, most patients spend the night after a sand bath, getting to know each other, sharing their experiences of the disease and sand bath, and supporting one another. These friendly conversations mean a great deal to them, as most of them strongly agree with the statement, “People do not take my medical condition seriously enough” on the Tampa kinesiophobia scale. Considering that multidisciplinary treatment is favored for rheumatic pain owing to its complexity, the therapeutic milieu used to treat kinesiophobia includes interpersonal communication, such as group sessions and feedback sessions.^[58]^

Siwa is a very dry, hot, and distant oasis in the middle of the desert, especially during the summer months. Traditionally, patients go there seeking for a relief from pain. The treatment protocol followed depends on the healer’s experience, and it needs to be assessed and standardized. Although the current study showed significant changes in all the parameters measured in the 5-days group, exposure time cannot be considered as a variable affecting the outcomes because of the insignificant difference found between the 2 groups’ outcomes. In the current study, the clinical impact of traditional Siwan therapy cannot be reported, and it is difficult to derive causal relationships because there is no control group.

Meanwhile, the nonsignificant differences in results between the groups can be attributed to the small sample size. However, Siwa is a distant place that can receive 70 patients per year. The nonsignificant differences in results might also be attributed to the short duration of the measurement because better improvement in RA patients was found 1 month after treatment,^[35]^ which is supported by the results of group two. This strongly suggests that more repeated measures studies are needed to investigate the optimum effects of this type of therapy.

*Limitation*: These research findings are limited to the applied traditional Siwan therapy program followed by the traditional healers in Siwa and the measured parameters. Further research with a larger sample size, control group, and long-term effects is required. More research is needed to compare the different outcomes and treatment protocols used in this study.

## 5. Conclusions

Despite the significant changes in central sensitization, pain severity, and pain threshold in the most painful joint after 3 and 5 days of traditional Siwan therapy, no significant difference was found between the 2 groups. All pain parameters produced favorable results after 5 days. Regarding the exposure duration of treatment and 3 and 5 days of exposure, there was no significant difference in the outcomes.

## Acknowledgments

The authors thank the patients who willingly participated in this study and extend their gratitude to the Siwan traditional therapy centers, Haj Sherif Senwsy Center, Haj Ahmed Mosa Center, Haj Abdelrahman Elshriak Center, Haj Setwhy Elwahy Center, Haj Ebrahim Zamour Center, Haj Omar Taqa Center, and Haj Ahmed Damdom Center for their cooperation. The authors appreciate the support provided by the Deanship of Scientific Princess Nourah bint Abdulrahman University Researchers Supporting Project number (PNURSP2023R206), Princess Nourah bint Abdulrahman University, Riyadh, Saudi Arabia.

## Author contributions

**Conceptualization:** Noha F. Mahmoud, Howida A. Fouda, Islam I. Omara, Nashwa M. Allam.

**Data curation:** Islam I. Omara.

**Formal analysis:** Howida A. Fouda, Islam I. Omara.

**Funding acquisition:** Noha F. Mahmoud.

**Investigation:** Noha F. Mahmoud, Howida A. Fouda, Nashwa M. Allam.

**Methodology:** Howida A. Fouda, Nashwa M. Allam.

**Project administration:** Howida A. Fouda.

**Resources:** Noha F. Mahmoud, Nashwa M. Allam.

**Software:** Islam I. Omara.

**Supervision:** Noha F. Mahmoud.

**Validation:** Howida A. Fouda, Nashwa M. Allam.

**Visualization:** Nashwa M. Allam.

**Writing – original draft:** Islam I. Omara, Nashwa M. Allam.

**Writing – review & editing:** Noha F. Mahmoud, Howida A. Fouda, Islam I. Omara, Nashwa M. Allam.
